# Draft Genome Sequence of *Defluviimonas indica* Strain 20V17^T^, Isolated from a Deep-Sea Hydrothermal Vent Environment in the Southwest Indian Ocean

**DOI:** 10.1128/genomeA.00479-14

**Published:** 2014-06-05

**Authors:** Lijing Jiang, Minnan Long, Zongze Shao

**Affiliations:** a State Key Laboratory Breeding Base of Marine Genetic Resources, Xiamen, China; b Key Laboratory of Marine Genetic Resources, Third Institute of Oceanography, SOA, Xiamen, China; c Key Laboratory of Marine Genetic Resources of Fujian Province, Xiamen, China; d School of Energy Research, Xiamen University, Xiamen, China

## Abstract

Here, we present the draft genome sequence of *Defluviimonas indica* 20V17^T^, which was isolated from a deep-sea hydrothermal vent chimney sample in the southwest Indian Ocean. The draft genome sequence contains 4,268,338 bp, with a G+C content of 66.33%.

## GENOME ANNOUNCEMENT

*Defluviimonas indica* 20V17^T^ (CGMCC 1.10859 =JCM 17871 =MCCC 1A01802) was isolated from a hydrothermal sulfide chimney sample in the Southwest Indian Ridge (1). The genus *Defluviimonas* was first proposed by Foesel et al. (2), which classified it within the family *Rhodobacteraceae*. Currently, the genus contains only three species, while strain 20V17^T^ is the first isolate from the deep-sea hydrothermal environment in the genus *Defluviimonas.*

The draft genome of 20V17^T^ was sequenced by Illumina paired-end sequencing technology at Beijing Genomics Institute (BGI) (Shenzhen, China). The reads were assembled using SOAP*denovo* (version 1.05), and 236 contigs were generated, with a maximum contig size of 155,100 bp and an *N*_50_ contig length of 39,530 bp. The assembled genome comprises 4,268,338 nucleotides with a G+C content of 66.33%. Gene annotation was carried out by the NCBI Prokaryotic Genomes Automatic Annotation Pipeline (PGAAP). A total of 4,190 genes were predicted, including 4,119 protein-coding genes, 42 tRNAs, and 4 rRNAs.

*D. indica* 20V17^T^ was able to oxidize reduced sulfur compounds as energy sources. RAST annotation also revealed that the draft genome contains sulfur oxidation genes (*soxRSVWXYZABCD*). Moreover, 11 genes encoding an [NiFe] hydrogenase were found in the draft sequence, indicating that strain 20V17^T^ might also be able to use H_2_ as an electron donor. The metabolic plasticity of *D. indica* 20V17^T^, which uses both molecular hydrogen and reduced sulfur compounds as energy sources, might represent a very useful strategy for living in deep-sea hydrothermal vent systems. The draft genome sequence of 20V17^T^ will enable further studies on the physiology and metabolic potential of this species, especially its roles involved in sulfur and H_2_ oxidation, which are major energy metabolisms in deep-sea hydrothermal vent environments.

### Nucleotide sequence accession numbers.

This whole-genome shotgun project has been deposited in DDBJ/EMBL/GenBank under the accession no. AYXI00000000. The version described in this paper is the first version, AYXI01000000.
